# Topographic Keratoconus Incidence in Greece Diagnosed in Routine Consecutive Cataract Procedures: A Consecutive Case Series of 1250 Cases over 5 Years

**DOI:** 10.3390/jcm13082378

**Published:** 2024-04-19

**Authors:** Anastasios John Kanellopoulos, Alexander J. Kanellopoulos

**Affiliations:** 1 Ophthalmology Department, LaserVision Ambulatory Eye Surgery Unit, 115 21 Athens, Greece; alexandrosjohnk@icloud.com; 2 Ophthalmology Department, NYU Grossman Med School, New York, NY 10016, USA; 3 School of Medicine, European University Cyprus, Engomi, Nicosia 2404, Cyprus

**Keywords:** keratoconus, incidence, corneal ectasia, cornea tomography, Scheimpflug corneal tomography, form fruste keratoconus

## Abstract

**Background**: Scheimpflug tomography has for many years been an integral part of our pre-operative assessment in cataract extraction. We retrospectively reviewed the incidence of topographic keratoconus and keratoconus suspicion in our routine cataract surgery population over 5 years. **Setting:** The Laservision Clinical and Research Institute, Athens, Greece. **Methods:** In 1250 consecutive cataract surgery cases in otherwise naïve eyes, accounting for years 2017 to 2021, we retrospectively evaluated preoperative Pentacam HR imaging. The cases already classified as keratoconus were included in group A. The residual cases were assessed by five different experienced evaluators (two ophthalmic surgeons and three optometrists) for topographic and tomographic keratoconus suspicion based on irregular pachymetry distribution, astigmatism truncation, and/or astigmatic imaging irregularity and included in group B. Regular corneas, by this assessment, were included in group C; irregular corneas, as determined by the evaluators but unrelated to keratoconus, were included in group D. **Results:** Based on the above, 138 cases (11.08%) were classified by Pentacam tomography as keratoconus and by default were included in group A. Of the residual cases, 314 or 25.12% were classified as suspect keratoconus and included in group B; 725 cases (58%) were classified as normal and non-keratoconus and included in group C; and 73 cases or 5.84% were placed in group D as non-keratoconus but abnormal. There was no disagreement between the five evaluators over any of the cases in groups C and D, and little variance among them for cases included in group B (less than 5% by ANOVA). **Conclusions:** The incidence of keratoconus and corneas suspicious for keratoconus in Greece appears to be much higher than respective reports from other regions: one in ten Greeks appear to have topographic keratoconus, most not diagnosed even by the age of cataract surgery, and almost an additional one in four may have suspicious corneal imaging for keratoconus. These data strongly imply that routine screening for disease should be promoted among Greeks, especially during puberty, to halt possible progression; moreover, careful screening should be performed when laser vision correction is being considered.

## 1. Introduction 

Keratoconus is considered an unpredictably progressive disease that biomechanically “softens” the cornea. Current scientific knowledge suggests that the human cornea is probably the only part of the human body that is fully developed at birth, making the human cornea a unique anatomical tissue in shape and structure [1,2,3,4].

Additionally, regarding keratoconus and corneal ectasia, current medical knowledge dictates that it may initially affect the biomechanical stability of the cornea, likely through corneal collagen properties and/or its parenchymal material properties, thus affecting the biological rigidity of the corneal collagen and/or the surrounding stromal infrastructure. It has yet to be determined whether this disease process is initiated by concomitant allergic etiology resulting from increased kinase and/or an enzymatic change in the corneal stroma and tear film microenvironment that, according to much of the literature, compels patients to rub their eyes during their sleep. Another speculative origin of this disease is an initial change in the cornea’s standard biomechanical properties and the initiation of keratoconus as a progressive cascade beginning with corneal ectasia disease; the initial “softening” or lessening of the biomechanical strength is followed eventually by stromal thinning that promotes a change in the cornea’s shape. Typically, inferior steepening is associated with respective superior corneal flattening that may somehow trigger eye rubbing and further thinning of the cornea stroma, followed by an inevitable increase in the posterior concave curvature of the cornea and eventually the anterior curvature of the cornea. The initial stages of change in anterior corneal shape are masked by epithelial remodeling. It may be that this process is the one that changes the kinase and or enzymatic environment, propagating eye rubbing as a further consequence of corneal ectasia. 

Whether “the egg creates the chicken or the chicken the egg” in this situation is uncertain [4,5,6,7,8,9,10,11,12,13,14,15,16,17,18,19,20,21,22,23,24,25,26,27,28,29,30].

Dynamic corneal epithelial remodeling appears to adapt to these changes, and its three-dimensional mapping may broaden our understanding and documentation of anterior curvature changes in the cornea in correlation with actual stromal thickness changes that take may place in progressive ectasia [22].

Our investigative team in Athens, Greece has, since 2003 and under an investigative protocol, used a prototype device (developed at the time by Priavision (Menlo Park, CA, USA)) for UV cornea cross-linking. They began using the device several years in advance of its formal approval for clinical use in the European Union, and the respective availability of a CE-marked commercially available device. All this investigative work subsequently reported in the peer-reviewed literature demonstrated drastic improvement in cornea rigidity as well as regression of all keratoconus corneal ectasia and keratometry indices. This work also marked the beginning of an exaggerated effort to explore the then boundaries of early clinical diagnosis, as CXL appeared to tangibly halt the progression of keratoconus in teenagers and young adults (thus halting the associated morbidity it carries). Additionally, our investigative team introduced the Athens protocol CXL technique to combine a therapeutic shape-changing excimer laser with surface ablation [31,32].

All these historical data are noted as an introduction the fact that the literature reports that 1 in 1000 to 2000 people in the general population suffer with keratoconus. Extensive screening assessments for keratoconus in young adults and teenagers, using corneal imaging instead of visual function morbidity alone, have produced an early speculative figure of 1 in 35 of patients in Athens, Greece, upon screening for laser refractive surgery using corneal imaging. The latter study displayed a high incidence of documented disease and even early signs of keratoconus upon imaging. Our investigative work also suggested a higher degree of familial correlation involved in keratoconus (close to 90%), while the other literature at the time reported a 10% genetic correlation. 

In this study, we focused on a very different patient population evaluated and treated by our ophthalmology department in Athens, Greece: we retrospectively evaluated a large group of consecutive cataract surgery candidates and the incidence of keratoconus and/or keratoconus suspicion evaluated by their corneal imaging captured as part of the IOL calculation process.

## 2. Methods

In this study, we used a retrospective review to evaluate consecutive cataract surgery cases performed in our center over the period of 5 calendar years. Cases with low endothelial cell density (ECD) (under 1500 cells/mm^2^) and/or significant endothelial cell polymegethism were excluded, as Fuchs’ endothelial dystrophy may affect the accuracy of corneal tomographic imaging. All prospective cataract cases were evaluated with ECD imaging as well as Scheimpflug tomography imaging (Pentacam HR, Oculus, Germany) as part of the IOL calculation protocol. When reviewing their data retrospectively, we were able to study the incidence of keratoconus both as determined by the Pentacam and additionally based on clinical suspicion raised by parameters in Pentacam imaging—mainly topographic cornea irregularity and cornea pachymetry irregularity— in this population [25,29,33,34,35].

We studied a group of consecutive cataract surgery cases; the group was composed of males and females evaluated and treated in our center in Greece, with an average of age 72 years (the range being 45 to 102 years old); they were previously unknown to our corneal and refractive surgery service that traditionally screens for keratoconus. 

Therefore, years later, we retrospectively studied a group that was theoretically irrelevant to keratoconus: cataract surgery candidates mainly in their seventies. 

This is a retrospective chart review study. All patients at the time of cataract surgery had provided consent for their procedure data to potentially be used in the future; it was to be anonymized for analysis, and this study adhered to the tenets of the Declaration of Helsinki.

All patients were Greek nationals covered by the National Health care system.

**Figure 1:**
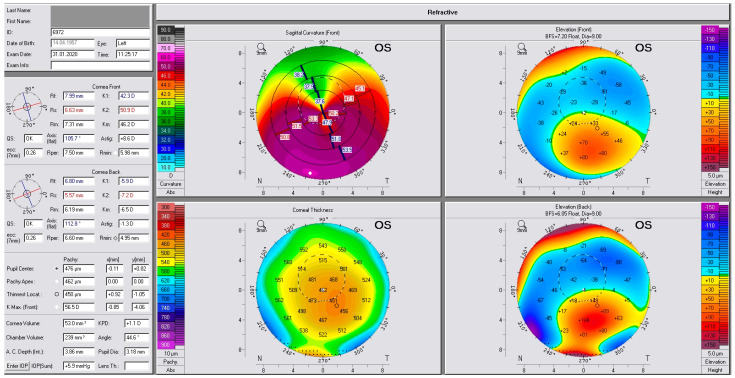
Illustrates the 4 maps refractive version of the Pentacam capture of a pre-operative case from our study subjects, for cataract that was never in the past diagnosed with keratoconus, now at the age of 63.

**Figure 2:**
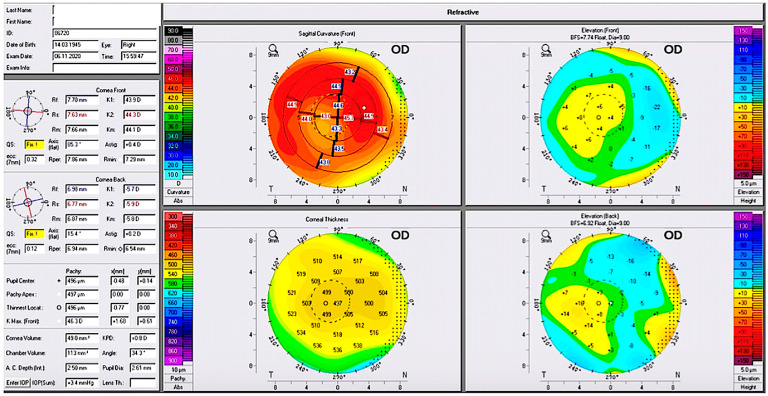
Illustrates the 4 maps refractive version of the Pentacam capture of another pre-operative case for cataract that was never diagnosed with keratoconus either, now at the age of 68.

As noted above, we excluded cataract surgery candidates with an endothelial cell density under 1500 cells/mm^2^ and/or polymegethism, as Fuchs’ endothelial dystrophy may affect the accuracy of Scheimpflug tomographic corneal imaging captured by the Pentacam. In the residual 1250 consecutive cataract surgery cases operated for cataracts and included in this review (all other cases were eyes naïve to corneal disease) accounting for the years 2017 to 2021, we retrospectively evaluated the topographic incidence of keratoconus. Beyond the number of cases classified by the Pentacam HR (stages 1 to 4), we additionally reviewed Pentacam images of all other cases for the suspicion of keratoconus. Based on irregular pachymetry distribution, as noted in the legends of Figure 1 and Figure 2 and including the following clinical assessment principles.

On the sagittal curvature map:

- astigmatism image truncation;
- and/or astigmatic irregularity as asymmetry between the two ends of the “bowtie”;
- and/or “scissoring” of the astigmatism “bowtie”.

On the total corneal pachymetry map,

- cornea pachymetry “step’ changes in ellipsoid instead of circular shape, which are usually “skewed” infero-temporally;
- more than two to three thickness “steps”; multiple steps (instead of the usual two or three) are unusual even in corneas with high cylindrical power.

Once these cases were grouped based on their imaging evaluation, the Scheimpflug imaging alone (for the non-keratoconus classified cases of group A) for each patient was made available; it was to be reviewed by five different experienced evaluators. These five evaluators were two ophthalmic surgeons and three experienced, cornea-fellowship-trained optometrists. All had extensive experience with several thousand patients’ tomographic corneal evaluations. 

The evaluated cases were stratified into one of four groups:

Group A: the keratoconus cases, classified as such by the Pentacam;

Groups B, C and D as classified by our expert reviewers:

Group B: suspicious for keratoconus based on the criteria noted above;
Group C: “normal” corneas for keratoconus both by the Pentacam and by the expert review;
Group D: irregular corneas not keratoconus-related and not noted in the patient’s documented respective ophthalmic history. The evaluation by these experienced clinicians was necessary to classify the Scheimpflug tomographic imaging, excluding those cases already defined by the device as keratoconus, into one of the other three different categories:

We additionally subsequently compared the evaluators’ data, looking at differences between the two surgeons and between the three optometrists, as well as comparing the opinions of the surgeons and the optometrists; we found percentages of agreement regarding groups B, C, and D using analysis of variance (ANOVA) statistics.

## 3. Results

Based on corneal tomographic data, the cases were classified using descriptive statistics of incidence, as follows:

A: A total of 138, or 11.04%, were included in group A by default, as they were found to be already classified by Pentacam analysis as keratoconus stages 1–3.
B: A total of 314, or 25.12%, were in group B, with suspected keratoconus or forme fruste keratoconus (as first introduced by Amsler).
C: A total of 725, or 58%, were placed in group C as they were found to have normal corneal parameters upon Scheimpflug tomography.
D: A total of 73, or 5.84%, were placed in group D, as they had non-normal features but no keratoconus.

Figure 1 illustrates the refractive versions of four Pentacam maps of the pre-operative phase of one of our subjects, now at the age of 63, who had cataracts but had not previously been diagnosed with keratoconus. This case was included in Group A by default as it was classified by the algorithm as stage 2 keratoconus based on the Amsler–Krumeich criteria. The tomography findings clearly illustrate corneal ectasia changes (which were never diagnosed until our assessment) in the upper and lower right images of anterior, posterior elevation; in the upper left image of sagittal total corneal power; and in the lower left image of the total corneal thickness map. Of special note here is the fact that cornea pachymetry changes are in an ellipsoid shape (non-circular), in multiple steps (instead of the usual two or three pachymetry step changes in corneas with high cylindrical power), and “skewed” infero-temporally, a finding that we have used in assessing suspect cases that are otherwise considered “non-keratoconus” by the algorithm used herein. 

Figure 2 illustrates the refractive versions of four Pentacam maps of the pre-operative phase of one of our subjects, now at the age of 68, who had a cataract that was never diagnosed with keratoconus. When all our evaluators assessed the anterior and posterior elevation data, the sagittal total corneal power data, and the thickness map, this case was considered a forme fruste (very early, subclinical) keratoconus case (using the criteria of thickness distribution noted in Figure 1). It was nevertheless classified as non-keratoconus and “normal” by the Amsler–Krumeich criteria-based algorithm used by the device.

**Figure 3:**
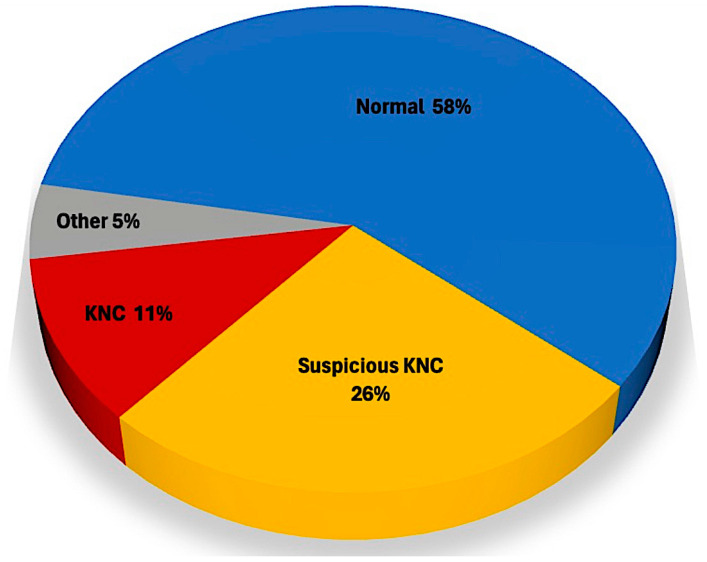
Pie chart with the distribution of our findings.

Figure 3 illustrates the pie graph with the data distribution of the findings. 276 There was no disagreement between the 5 evaluators for any of the cases in groups C and D, and little variance among them for cases included in group B (less than 5% using ANOVA).

There was no disagreement between the five evaluators over any of the cases in groups C and D, and little variance among them for cases included in group B (less than 5% using an ANOVA).

There was agreement of 99% between the surgeons and the same between the optometrists as well, suggesting that the methodology used was accurate as far as our criteria in selecting the cases that were keratoconus suspect or otherwise named forme fruste keratoconus. These was no difference in age, sex between the 4 groups studied (ANOVA of the mean age for each group).

## 4. Discussion

The incidence of keratoconus and corneas suspicious for keratoconus in Greece appears in our data to be far higher than in all other respective reports: one in ten of the patients evaluated in our study appeared to have topographic keratoconus (the vast majority not diagnosed even by the age of cataract surgery), and almost an additional one in four were found to have irregular corneal imaging suggesting forme fruste keratoconus.

These data are in stark contrast to some of the literature suggesting a keratoconus incidence level of 1 in 2000 persons in the general population.

Our research team has reported in several publications that close to 1 in 35 of our patients displayed clinical or subclinical keratoconus, and that number may have been 1 in 10 (based on clinical experience) when taking into account patients from neighboring Cyprus, as Greek–Cypriot individuals have a significantly similar genetic background [34]. Our research has also documented a higher degree of familial correlation in keratoconus than previously reported in the literature. The previously reported 10% genetic correlation has been revisited in our research documentation, which showed a potential peak of 90% correlation in patients with documented keratoconus appearing to have at least one parent—mother and/or father—that also demonstrated topographic or tomographic suspicion of keratoconus upon tomographic corneal imaging. 

Our team has reported in the past that as cornea diagnostics evolve, the incidence of keratoconus in the general population, as noted in existing studies, has turned out to be much higher than 1 in 1000. We have reported that the actual incidence of keratoconus may be closer to 1 in 35 in the Greek population that our scientific and clinical team studied [33,34,35,36].

As documented in the literature, most of the human body undergoes drastic development from childbirth; nevertheless, the cornea appears to be fully developed—at least in its typical dimensions, diameter, epithelial thickness, clarity, and curvature—at birth. Recent scientific reports have documented that the development of diagnostic tools for accurately assessing the biomechanical properties of the cornea is still in progress [37]. 

Corneal thickness and curvature diagnostics have been well documented in the literature to offer early keratoconus diagnosis findings. This is of significant clinical importance, particularly as in the last two decades, the advent of CXL has offered stabilization of keratoconus, and its early diagnosis and treatment significantly limits associated morbidity. Clinical research that has been reported and is noted above has shown the capacity to accurately document corneal changes, mainly through curvature cornea imaging or anterior segment OCT.

A potential bias to these data is the fact that our practice is a reference center for the diagnosis and treatment of keratoconus on both a national and international level. Nevertheless, the keratoconus patients that we evaluate in our cornea/refractive surgery department are usually younger adults and/or adolescents in their second to fourth decade of life. Additionally, they demonstrate a very uneven gender balance, with men being almost six times more common than women. The actual group of patients evaluated in this study was, by definition, irrelevant to keratoconus as an initial clinical diagnosis (or even suspicion), as these patients were evaluated and treated by our cataract surgery service and not the cornea/refractive surgery service in our center. The studied patients were over 60 years of age, usually in their seventies, and there was equal ratio of men and women. They were evaluated for the chief complaint of progressive and recent visual changes; through our evaluation, these visual changes were documented to relate to significant cataract formation, thus indicating a cataract procedure for ophthalmic management. The cataract surgery service and intraocular lens calculation protocol in our institution includes ECD measurement and Pentacam imaging (among the other standard diagnostics), and these data provided the basis for a retrospective review of the tomography data that were used and analyzed in this study. 

Thus, one can assume that the conduction of the study was blinded towards keratoconus diagnosis in its original data collection method, which was cornea tomography measurements on patients planned to undergo cataract surgery in our institution over the period of five years. Nevertheless, we realize that in evaluating and treating a keratoconus patient, it is possible that an elder member of that keratoconus patient’s family may seek ophthalmology services from our institution when their visual function is challenged by cataract formation. This point does carry some potential bias regarding the large group of cases evaluated, who may have had a slightly higher incidence of keratoconus and keratoconus suspicion than the general population in Greece, as noted above. 

Nevertheless, in searching reported investigations of the incidence of keratoconus, we found that the consecutive assessment pre-operative data from cataract procedures (which include both female and male patients), especially when they are not selected based on cornea pathology but instead are selected based on how their recently deteriorating visual function proved to be affected by cataracts’ formation, may provide a new perspective on the current significant higher incidence of keratoconus among the cataract surgery-aged population in Greece when Scheimpflug tomography imaging is used and interpreted by expert clinicians as a criterion. Also open for discussion is our keratoconus suspicion criteria, which may be broader than the criteria that are currently underlined in the literature. The tomography device we used, the Pentacam HR, has an intrinsic classification system based on an algorithm established by the manufacturer (Oculus) and named the Amsler–Krumeich keratoconus classification. It ranges from nil (no keratoconus detected) to keratoconus between stages 1 to 4, then to stage 4 (which represents the most significant documented keratoconus corneal irregularity in terms of curvature change and therefore ectasia advancement). Most Pentacam tomography device models also feature an additional algorithm, the Bellin–Ambrosio cornea thickness change curve criterion, which may also function as a screening tool for keratoconus diagnosis. We did not use this criterion in our assessment.

Our team has reported in the past that keratoconus diagnosis criteria may need to be revisited, as pointed out by several of our research data outlined below:

1. With regard to corneal tomography, the IHD (index of height decentration) seems to be the most sensitive parameter for documenting corneal regularity or change and the evolving irregularity that corneal ectasia typically manifests. The second is the ISV (index of surface variance), according to the extensive correlation of the studied keratoconus cases as well as the topometric asymmetry indices, provided by the Pentacam. The third is the IHA (index of height asymmetry) [33,34]. All are far more sensitive than visual acuity, a facet of visual function and metric that usually brings a corneal ectasia patient to ophthalmic care. Ironically, in our study, visual acuity was a very poor predictive and diagnostic factor for keratoconus, meaning many clinicians should be cautioned against using it as a corneal imaging methodology and a broad screening method for the diagnosis of keratoconus in the general population. These anterior corneal curvature asymmetry indices are provided by the topometric data analysis that the Pentacam device itself can provide.
2. Also referenced in the study is our extensive reported work in evaluating corneal epithelial mapping changes, both with high-frequency ultrasound and anterior segment OCT, as an early sign of ectasia development and its respective clinical importance in early diagnosis. This parameter was not used in this study as we did not have pre-cataract surgery images of the cornea and epithelia of all patients that we retrospectively analyzed [33,35].
3. Lastly, we have also reported a suggested algorithm using anterior segment OCT, derived corneal thickness, asymmetry measurements as an early sign of keratoconus diagnosis, and even the suspicion of keratoconus [35].

The data provided by our study are compelling, underlining that one in three Greeks either have topographically diagnosed keratoconus or are highly suspicious for keratoconus (which we may refer to as forme fruste keratoconus), the vast majority without ever knowing it or being diagnosed, considering these patients are mostly elderly. This confirms speculations noted in our previous publications that the incidence of keratoconus in Greece is likely much higher than what was initially thought. These data represent a significant database for screening families. A person in his or her seventies being diagnosed with keratoconus or forme fruste may not be significantly affected any further (besides the challenge in calculating IOL for their cataract surgery). However, the diagnosis may raise concerns for younger members of their family, who may want to be screened for keratoconus, especially if there are grandchildren who are teenagers or in their early twenties. Potential effective early keratoconus screening for these potential patients, prior to the disease becoming symptomatic, may prove pivotal in offering treatment when needed and potentially avoiding clinically significant disease progression, as a strong preventive-medicine measure. 

The data presented herein appear compelling and may justify routine screening for keratoconus in the Greek population, especially in puberty, so that diagnosis can happen earlier. 

Proper screening may help us in lieu of CXL, which has become the focus of care globally in attempts to eradicate clinical keratoconus altogether; it may theoretically minimize the need for cornea transplantation, which remains the major, if not the main, indication for cornea transplantation.

In conclusion, the data introduced herein strongly suggest routine screening for this disease among Greeks, especially in puberty, and strong clinician caution regarding keratoconus or cornea irregularities that suggest keratoconus predisposition when screening for laser vision correction (especially LASIK and/or Smile).

## 5. Conclusions

The incidence of keratoconus and corneas suspicious for keratoconus in Greece appear to be significantly higher than in reports from other regions: one in ten Greeks appear to have topographic keratoconus, most not diagnosed even by the age of cataract surgery, and almost an additional one in four may have corneal imaging that is suspicious for keratoconus. These data strongly imply that routine screening for disease should be promoted among Greeks, especially during puberty, to halt possible progression; moreover, careful screening should be performed when laser vision correction is being considered.

## Data Availability

All data will be available on the institution website: www.laservision.gr/KCN cataract study.
